# Effects of muscarinic and nicotinic receptors on contextual modulation in macaque area V1

**DOI:** 10.1038/s41598-021-88044-7

**Published:** 2021-04-16

**Authors:** Jose L. Herrero, Alexander Thiele

**Affiliations:** 1 Feinstein Institute of Medical Research, New York, USA; 2 Biosciences Institute, Newcastle University, Henry Wellcome Building, Newcastle upon Tyne, NE2 4HH UK

**Keywords:** Cellular neuroscience, Visual system

## Abstract

Context affects the salience and visibility of image elements in visual scenes. Collinear flankers can enhance or decrease the perceptual and neuronal sensitivity to flanked stimuli. These effects are mediated through lateral interactions between neurons in the primary visual cortex (area V1), in conjunction with feedback from higher visual areas. The strength of lateral interactions is affected by cholinergic neuromodulation. Blockade of muscarinic receptors should increase the strength of lateral intracortical interactions, while nicotinic blockade should reduce thalamocortical feed-forward drive. Here we test this proposal through local iontophoretic application of the muscarinic receptor antagonist scopolamine and the nicotinic receptor antagonist mecamylamine, while recording single cells in parafoveal representations in awake fixating macaque V1. Collinear flankers generally reduced neuronal contrast sensitivity. Muscarinic and nicotinic receptor blockade equally reduced neuronal contrast sensitivity. Contrary to our hypothesis, flanker interactions were not systematically affected by either receptor blockade.

## Introduction

The perception of an image element is strongly influenced by the spatial context embedding the image element itself^1,2^. This influence can lead to suppression or facilitation depending on the specifications of the context. At the perceptual level facilitation occurs when a low contrast Gabor target presented foveally is flanked by collinear Gabor elements^3–5^. Facilitation is most pronounced if flanking stimuli are presented at a distance of 2–4 times the wavelength (*λ*) of the central Gabor^6–8^. When flankers are closer to the target (< 2*λ*) suppressive influences dominate, particularly for high contrast target stimuli or non collinear flankers^9,10^. However, flanker induced inhibition has also been reported for larger wavelength (distance) in macaque parafoveal locations^11^.

These contextual effects have been argued to arise from lateral neuronal interactions in striate cortex^12–15^, but equally from feedback connections from higher areas^14–19^. V1 neuronal responses to a central target can increase in the presence of collinear flankers^20–22^. This is most pronounced for low contrast targets^22,23^. However, a more recent study has argued that the flanker facilitation is an effect of flanker intrusion into the neuron's receptive field, which results in overall reduced contrast sensitivity^11^. In addition to lateral interactions, feedback influences surround integration in primary visual cortex^15,16,18,19,24–29^.

The efficacy of both, lateral and feedback connections is regulated by a variety of neuromodulators. In vitro and in vivo studies have demonstrated that the neuromodulator acetylcholine (ACh) can affect lateral interactions in the cortex^30–32^. ACh reduces the efficacy of intracortical interactions via a muscarinic (M2) mechanism^30–33^, while it also increases the efficacy of feed-forward connections through nicotinic receptors^30,34–36^. However, muscarinic activation by ACh in cortex may have more complex effects on overall integration, due the expression profile of M1 and M2 type receptors on different cell types (for review e.g.^33^). We hypothesized that muscarinic blockade, increasing lateral and feedback interactions, will facilitate flanker-induced effects while reducing neuronal activity and contrast sensitivity^37–39^. Conversely, nicotinic receptor blockade (located pre-synaptically on thalamocortical input^34,35^) should result in a general gain decrease at all contrasts, without affecting flanker modulation.

Here we investigate the role of muscarinic and nicotinic ACh receptors (mAChRs & nAChRs) in flanker induced contextual modulation of V1 neurons in awake, passively fixating macaque monkey. Only a small fraction of cells showed flanker-induced facilitation. In most cells flanker-induced suppression occurred. Irrespective of the sign of these effects, muscarinic receptor blockade had no systematic effect on flanker interactions, as it could lead to increased as well as reduced flanker induced changes to contrast sensitivity. Equally, while nicotinic blockade reduced overall neuronal gain it had no systematic effect on flanker induced alterations to contrast sensitivity.

## Materials and methods

All procedures were approved by the Newcastle University Animal Welfare Ethical Review Board (AWERB) and carried out in accordance with the European Communities Council Directive RL 2010/63/EC, the US National Institutes of Health Guidelines for the Care and Use of Animals for Experimental Procedures, and the UK Animals Scientific Procedures Act. We used three adult awake male macaques (*Macaca mulatta*, age 6–10 years, weight 11–15 kg). Animals were motivated to engage in the task through fluid control at levels that do not affect animal physiology and have minimal impact on psychological wellbeing^40^. The study was carried out in compliance with the ARRIVE guidelines.

### Electrophysiological recordings and behavioural procedures

**Figure 1:**
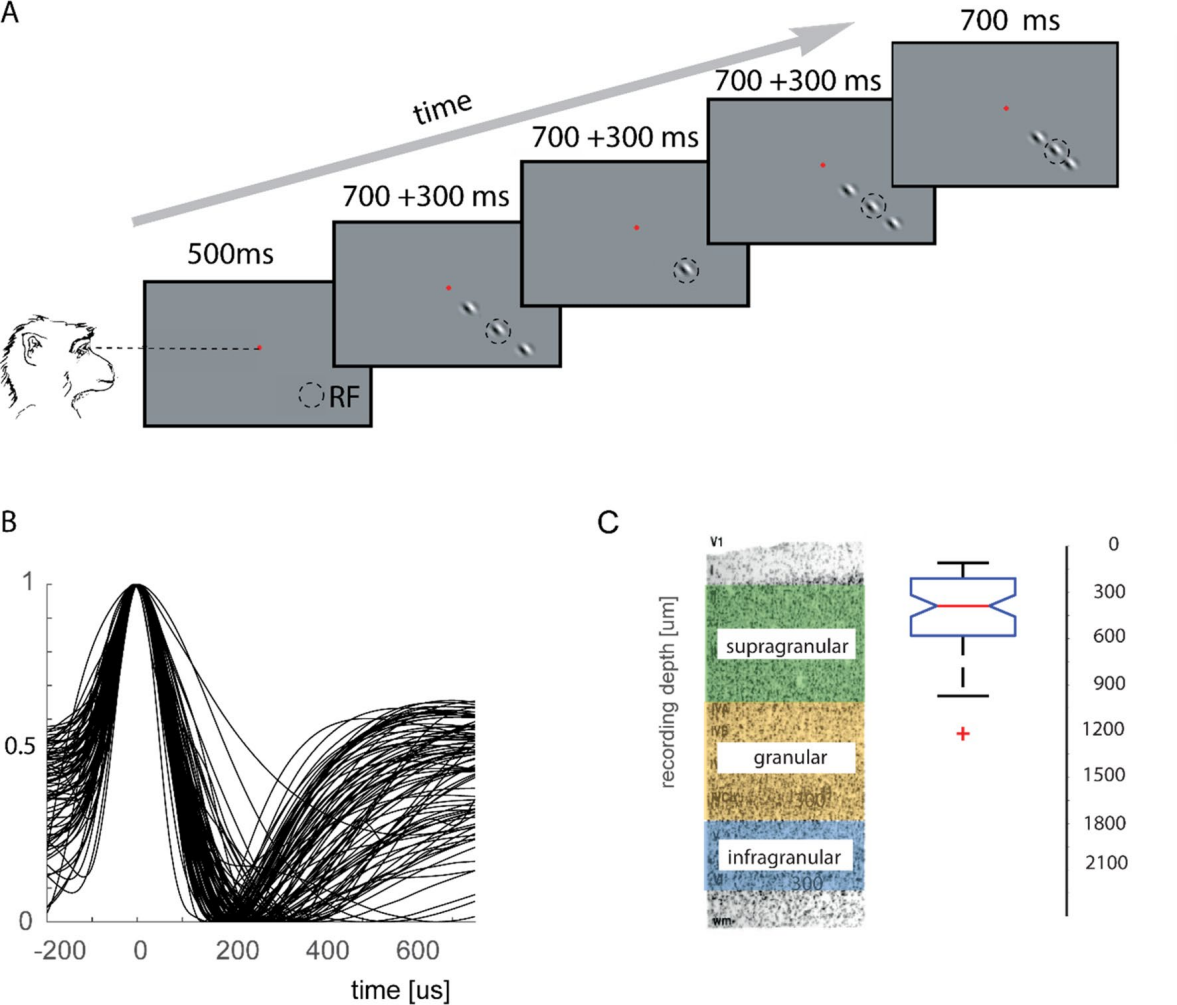
(**A**) The fixation task. Monkeys fixated a central point (red) and passively viewed a sequence of 4 stimuli displays. Each display consisted of a central target Gabor presented in the RF (dashed circle) of the neuron under study, either in isolation or together with two collinear flankers. Each display lasted 700 ms, with a 300 ms blank interval. Note that the circle outlining the RF is for illustrative purposes and was not part of the stimulus display. (**B**) Normalized mean waveforms of the cells recorded. (**C**) Median, quartile, and range of recording sites in V1, reconstructed according to depth records in relation to first spiking activity.

We recorded neurons in three male macaque monkeys (*Macaca mulatta*, 8–12 years of age). After initial training, monkeys were implanted with a head holder and recording chambers above V1 under general anaesthesia and sterile conditions (for details of surgical procedures, see^41^). Single-cell discharges were recorded extracellularly using a tungsten-in-glass electrode flanked by two pipettes^41,42^. Drugs were applied iontophoretically through these pipettes using the NeuroPhore BH-2 System (Digitimer). Stimulus presentation and behavioral control were managed by Remote Cortex 5.95 (Laboratory of Neuropsychology, National Institute for Mental Health, Bethesda, MD). Neuronal data was collected by Cheetah data acquisition (Neuralynx) interlinked with Remote Cortex. The waveforms of all spikes that exceeded a threshold set by the experimenter were sampled at 32 kHz. Offline sorting of these spike samples was carried out based on waveform features (Neuralynx spike sorting software, Version 2.51). Average waveforms of recorded cells are shown in Fig. 1B. While we aimed to isolate single cells, on occasions these may be somewhat contaminated by spikes from additional cells, as is generally the case with extracellular recordings. Recordings were performed once the first isolated spikes were encountered after crossing the dura. This was usually the case within 100–300 μm after the first background hash activity occurred. Once a recording was finished and the animal continued to work we advanced the electrode pipette by at least 150 μm, until we encountered the next well isolated spike, and performed the next recording. In rare cases we performed a third recording on a recording day. Thus, almost all out recordings were performed within the first 1000 μm of crossing the dura, and therefore most likely within the supragranular layers (Fig. 1C).

Monkeys were trained to keep fixation (eye window 1.2° in diameter) while a small oriented Gabor was presented in the periphery of their visual field, with or without two collinear flankers (Fig. 1). The fixation point (FP, 0.1° diameter) was presented centrally against a grey background (21 cd/m^2^) on a 20″ analogue cathode ray tube monitor (100 Hz, 1,600 × 1,200 pixels, 57 cm from the animal). Eye position was recorded with an infrared based camera system (Thomas Recording GmBH) and sampled at a rate of 250 Hz.

A trial started as soon as the monkey’s eye position was within a fixation window centred on the fixation point. After 500 ms four Gabor stimuli, matching the preferred orientation and spatial frequency of the neuron, were presented in succession. Stimulus presentation time was 700 ms followed by 300 ms gaps between presentations (details about the stimuli are given below). At the end of the four presentations, the fixation point disappeared and monkeys were rewarded if their eye position had been within the fixation window for the trial duration. If the monkey broke fixation before the FP disappeared the condition was repeated later in the block.

### Stimuli

Details of stimuli have been described before^11^. We copy these description here for ease of access. Stimuli consisted of either central Gabor elements presented in isolation or flanked by two iso-oriented flankers. The orientation and spatial frequency of the Gabors matched the neuron’s preference (see below). Each Gabor moved within a Gaussian aperture at 4 Hz temporal frequency. The motion was perpendicular to the Gabor’s orientation, and reversed direction at a frequency of 4 Hz. Within the sequence of 4 presentations per trial the order of stimulus presentation within a trial and between trials was randomised.

Central and flanker Gabors were identical in all respects except for their contrasts. The contrast of the central Gabor was varied between 0, 4, 8, 12, 16, 24, 32, and 64% (Michelson contrast), while the contrast of the flanking Gabors was fixed at 48%. The distance between the central and flanking Gabors could be 1, 2 or 3–4 times the Gabor wavelength (*λ*). The exact wavelength varied slightly with the spatial frequency preference. High spatial frequencies of for example 6 cyc/° would result in centre flanker distances of 0.166° at a distance of 1*λ*, whereby the receptive field centre would be filled by flankers. To account for this we used distances of 1.5, 2.8, and 4*λ* for spatial frequencies of ≥ 6 cyc/°, and distances of 1, 2, and 3*λ* for spatial frequencies of < 6 cyc/°. The large majority of our neurons preferred spatial frequencies of ≤ 4 cyc/° and only 8 neurons were measured with ≥ 6 cyc/°. We included them in our overall sample and treated them as if the wavelengths had been 1, 2, and 3*λ*, respectively. We also scaled the size of our stimuli, whereby the half width at half height of the Gaussian envelope was 0.3 times the spatial frequency for spatial frequencies of < 2 cyc/°, it was 0.4 times the spatial frequency for spatial frequencies between 2 cyc/° and 4 cyc/°, 0.5 times the spatial frequency for spatial frequencies between 4 cyc/°, and 6 cyc/°, and 0.6 times the spatial frequency for spatial frequencies of ≥ 6 cyc/°.

Twenty trials per stimulus, contrast, and drug application conditions were recorded in most recordings. Cells were excluded if fewer than 10 trials per stimulus, contrast, and drug application conditions were available.

### Receptive field characterization

For each recording site we initially determined the location of the receptive field (RF) as well as the optimal orientation, spatial frequency and phase using reverse correlation techniques^43,44^. Briefly, the location of the RF was estimated by mapping the classical receptive field with briefly presented dark and light squares (0.1° width, 100% contrast) at pseudo-random locations on a 10 × 10 grid (a 1 × 1° area). The RF centre was taken as the location of the peak of the Gaussian fitted to the response distribution^25^. The mean RF eccentricity was 3.25°, 5.6°, and 5.7° in monkeys 1, 2, and 3, respectively. Thereafter, the tuning properties were estimated using static sinusoidal gratings (1° diameter) centred on the RF. These gratings varied in orientation (12 orientations, 0–165°), spatial frequency (1, 3, 5, 7, 8, 9 or 10 cycles/°) and phase (0, 0.5π, π, 1.5π) every 60 ms in a pseudo-randomized order. The stimulus that yielded the peak response was taken to represent the preferred orientation, spatial frequency, and phase of the neuron under study. The obtained parameters were used to determine the spatial frequency and orientation of the central and the flanking Gabor elements, which had identical properties. These reverse correlation procedures were conducted while monkeys fixated centrally on the CRT monitor.

### Drug application procedures

The opening diameter of the pipettes varied between 1–4 μm and their resistance varied between 10–150 MΩ, with most recordings (> 90%) at 20–80 MΩ^42^, while recording electrode resistance was 0.5–2 MΩ. Hold currents for scopolamine and mecamylamine were usually − 10 nA, in rare occasions (when the pipette resistance was 10–20 MΩ) it was − 40 nA. Pipette electrode combinations were inserted into V1 through the dura on a daily basis without the use of guide tubes. The integrity of the electrode and the pipettes were checked under the microscope before and after the recording sessions, in addition to measurements of the pipette impedance made before and after the recording at each recording site. The details regarding drug concentration, pH and application current were: scopolamine (0.1 M, pH 4.5, median current strength: 40 nA, 25 percentile: 30 nA, 75 percentile: 50 nA), and mecamylamine (0.1 M, pH 4.5, median current strength: 10 nA, 25 percentile: 5 nA, 75 percentile: 10 nA).

Drug application was continuous during blocks of ‘drug applied’. The duration of each block could vary depending on the number of repetitions for each condition that we aimed for, and depending on the number of eye fixation errors that the monkey made. On average drug application for each block was ~ 7–12 min. For the data analysis we removed the first 2 trials of each condition from the data set, as drug effects and recovery usually occur with a slight delay of ~ 30 s. We regularly compensated for the change in current during the ejection condition by increasing the hold current of one of the two pipettes, thereby keeping the overall current identical between the ‘hold’ and ‘eject’ conditions.

### Analysis of the physiological data

Parts of the analysis methods have been described before^11^. First, we looked at trials before any drug was applied (i.e., trials during the baseline period). We tested whether the contrast of the central Gabor or the presence of flankers significantly affected neuronal activity, and whether there was a significant interaction between these factors (ANOVA, p < 0.05). We used the response period from 200 to 700 ms after stimulus onset for our main analysis. This involves mainly the sustained steady-state response. However, separately, we also analysed the response period from 50 to 200 ms to determine how flankers and cholinergic antagonists affect the transient response period. Neurons were analysed further if contrast and flanker presence significantly affected firing rates, or if a significant interaction between contrast and flanker occurred (ANOVA, p < 0.05). A total of 119 out of 124 cells (48, 19, and 52 from monkey 1, 2, and 3, respectively) passed the basic statistical test (two-factor ANOVA: factor 1, contrast; factor 2, flanker presence). For the initial recordings we used two target-flankers spatial distances (1 and 3*λ*) instead of three (31 cells out of 67 cells, all recorded in monkey 1), but in our later recordings we used three flanker distances to increase the flanker-distance sampling density. Because of this difference we exclusively focus on target-flankers spatial distances (1 and 3*λ*) and on the no flanker condition.

The cells that passed the basic statistical test (119/124) were tested for drug effects (three-factor ANOVA: factor 1, contrast; factor 2, flanker presence, factor 3, drug application). Neurons were analysed further if drug application significantly affected firing rates, or if a significant interaction between drug and contrast/flanker occurred (ANOVA, p < 0.05). A total of 108 cells passed the drug test (ANOVA, p < 0.05). We included cells were at least one control, one drug applied and one recovery block was recorded, and where the activity during the initial control and the recovery block did not differ significantly (ANOVA, p > 0.05). A total of 83 out of 108 neurons exhibited a significant drug effect and good recovery after drug application and were subjected to further analysis. From these, 48 were tested with scopolamine and 35 with mecamylamine.

We quantified the target-evoked response for each contrast level *c* in the presence and absence of flankers under control and drug-applied conditions. The responses for the control and recovery period was combined and compared to the drug induced activities. The response to the target stimulus (*R*_*Target*_(*c*), centre Gabor) was calculated as the difference between the response when target and flankers were present (*R*_*Target*+*Flankers*_(*c*)) and the response when only the flankers were present (*R*_*Flankers*_). For the condition where no flankers were presented we treated the zeros percent contrast target condition as the flanker present (*R*_*Flankers*_) condition. This resulted in:

$$R_{Target} (c) = R_{Target + Flankers} (c){-}R_{Flankers} .$$

To determine the effect of the flankers on contrast tuning in the presence and absence of drug, contrast response functions were obtained for each neuron in both drug conditions for each flanker wavelength separately. Responses to the target only (i.e. no flanker condition) were also collected and used as reference. Each contrast response function was based on the *R*_*Target*_(*c*) to 10–30 repetitions of each contrast and a total of 80–240 stimulus repetitions for each flanker condition and drug state (control, drug-applied, and recovery). Contrast response functions were fitted for each flanker condition with a hyperbolic ratio function of the following form:

1 $$R_{{{\text{target}}}} (c) = \, R_{{{\text{target(max)}}}} *(c^{n} /\left[ {c^{n} + c50^{n} ]} \right) + M$$

where *R*_*target(max)*_ is the saturated response, *c50* is the contrast at which the half maximal response is reached, *n* determines the slope of the contrast response function, and *M* corresponds to the spontaneous activity. This model provides a good approximation of contrast response functions in monkey visual cortex^45–47^, and we used multidimensional constrained nonlinear minimization (Nelder-Mead) to minimize the summed squared difference between data and model (Matlab 19b, Mathworks), whereby the constraint was that c50 could not exceed a contrast of 80%.

### AUROC analysis of neuronal activity

Strengths of flanker modulation were quantified by calculating the area under the receiver operating characteristic (AUROC) curve on the basis of single-trial responses. AUROC values of 0.5 indicate that an ideal observer can only perform at chance level in deciding the contrast of the target Gabor as being different from the background contrast. Higher AUROC values indicate greater contrast response enhancement, thus if the presence of flankers increased contrast detection, AUROC values should be increased in flanker-present condition relative to flanker-absence condition. Likewise, if scopolamine or mecamylamine decreased contrast detection, AUROC values should be decreased in the presence of drug compared to its absence. After calculating AUROC values for each contrast level (in each flanker condition under the presence or absence of drug), we fitted a Weibull function to each set of eight AUROC values. The point in time that the fitted function reached 82% of its maximum was defined as neuronal threshold. Neuronal thresholds were calculated for each neuron, drug, and flanker condition. Neurons with lower thresholds in no-flankers compared to flankers condition (*Theshold*_*without flankers*_ < *Threshold*_*with flankers*_) were considered to be suppressed by the flankers, while neurons with *Theshold*_*without flankers*_ > *Threshold*_*with flankers*_ where considered to be facilitated. To determine whether suppression or facilitation was significant for individual cells (i.e. whether the threshold differed significantly when the flankers were introduced; in both control and drug conditions), we fitted each function independently with the Weibull function and determined the chi-square error of the individual fits, and also when fits were forced to obtain the same *threshold*. The difference of the chi-square errors for the two approaches can be used to test whether the parameter of interest significantly changes when flankers are presented^48–50^. This procedure was also used to test whether the threshold changed when the drugs were applied.

Variables from the fitting procedures (R_max_, c50, thresholds and slopes) were used to derive a modulation index (MI): modulation index = (*Var*_*without flankers*_ – *Var*_*with flankers*_)/(*Var*_*without flankers*_ + *Var*_*with flankers*_).

Whereby Var is the variable of interest.

## Results

We have previously shown that flanker presentation equivalent to the one used here results in mostly inhibitory interactions in macaque V1^11^. This was the case even at low contrast and at flanker distances reported to cause facilitation in cat visual cortex and human psychophysics^7,8,22^. In the context of this paper we are interested how cholinergic blockers affect flanker modulation. We thus focus on cells where cholinergic blocker application significantly affected neural activity (Methods for details).

### Single cell examples of collinear flanker effects for nicotinic and muscarinic blockade

**Figure 2:**
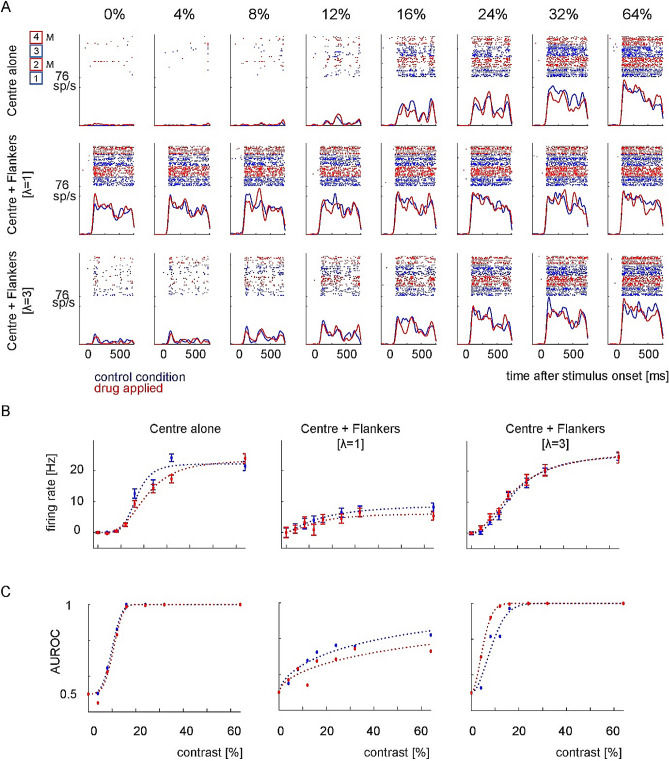
Effects of mecamylamine and collinear flankers on a representative sample neuron. (**A**) Single trial responses (raster plots) and peri-stimulus time histograms (PSTHs) of an example neuron during control (blue) and drug-applied (red) conditions. Each column shows a different contrast (indicated above the column) of the central Gabor (flanker contrast was fixed to 48%), and each row a different flanker condition. First we recorded responses when mecamylamine was not applied (blue rasters histograms, symbols and lines, Box 1), followed by responses when mecamylamine was applied (red rasters histograms, symbols and lines Box 2, M indicates mecamylamine application), a recovery period (Box 3), and another mecamylamine applied period (Box 4). The first 2 trials from each block are not shown as they were excluded from the analysis. The appearance of a central Gabor elicited a response that increased with the contrast of the stimulus. Mecamylamine generally decreased the responses. In the absence of a central Gabor (0% contrast), flankers at closer distances increased responses to a greater extent than flankers at longer distances due to intrusion in the receptive field. sp/s = spikes per second. (**B**) Contrast *R*_*target*_ functions of the cell shown above in the absence (blue) or presence (red) of scopolamine for the different flanker conditions. Mecamylamine decreased Rmax for the flanker distance of 1λ, and it increased c50 when no flankers were present. (**C**) contrast response functions assessed using AUROCs. Mecamylamine had no effect on contrast sensitivity when no flankers were presented, it increased the threshold at a flanker distance of 1λ, but decreased the threshold at a flanker distance of 3λ.

Figure 2A illustrates the response of an example neuron to different contrasts during control (blue) and drug-applied (red) conditions. We recorded the effect of flankers on firing rates when mecamylamine was not applied (10 trials, box 1). Thereafter we recorded the effect of the flankers when mecamylamine was applied (11 trials; box 2), followed by recovery (14 trials; box 3), and another block with mecamylamine applied (9 trials, box 4). In the absence of the flankers and without central stimulation of the receptive field (0% contrast stimulus), the neuron’s firing rate remained at the level of the spontaneous activity. As expected, the appearance of a central Gabor elicited a response that increased with the contrast of the stimulus (F(7,1444) = 264,0.7, p < 0.001, 3 Factor ANOVA: contrast, drug, flankers). Mecamylamine decreased the response at all contrasts (F(1,1444) = 5.62, p = 0.018). The presence of the flankers also had a main effect on the response of this neuron (F(3,1444) = 392.44, p < 0.001). There was a mild trend towards an interaction between flankers and drug (flankers*drug; F(3,1444) = 2.3, p = 0.078), and there was an interaction between flankers and contrast (flanker*contrast; F(21,1444) = 15.45, p < 0.001).

When two collinear flankers were presented without the central target (*R*_*Flankers*_), the neuron’s responses often increased above the spontaneous activity level. This response was significant for both flanker distances (1*λ* and 3*λ*; *p* < 0.001 and *p* = 0.001 respectively, two sided rank sum test). These effects did not depend on whether mecamylamine was applied (drug *R*_*Flankers*_ vs spontaneous at 1*λ* and 3*λ*; *p* < 0.001 and *p* = 0.041, two sided rank sum test). For this example cell R_max_ remained similar between the control and the drug condition when center alone stimuli were presented (Fig. 2B; R_max(control)_ = 22.19 Hz, R_max(drug)_ = 23.48 Hz), but c50 increased with drug application (c50_(control)_ = 16.1%, c50_(drug)_ = 20.2%contrast). Thresholds as assessed by AUROC (Methods) did not change significantly (Fig. 2C; p = 0.39; threshold_(control)_ = 11.04%, threshold_(drug)_ = 11.62% contrast). At a flanker wavelength of 1*λ*, Rmax was overall reduced, but this was more pronounced upon drug application(R_max(control)_ = 9.41 Hz, R_max(drug)_ = 5.92 Hz). C50 was overall increased by flankers of 1*λ*, but they were similar between control and drug application (c50_(control)_ = 15.1% contrast, c50_(drug)_ = 13.8% contrast). Thresholds as assessed by AUROC (methods) were much higher compared to the no flanker condition, but did not change significantly between control and drug condition (p = 0.21; threshold_(control)_ = 50.1, threshold_(drug)_ > 80% contrast). At a flanker wavelength of 3*λ*, Rmax was overall increased relative to the no flanker condition, with little difference between control and drug application(R_max(control)_ = 25.8 Hz, R_max(drug)_ = 26.6 Hz). C50 was slightly higher for the flanker 3*λ* condition than the no flanker condition, but similar between control and drug application (c50_(control)_ = 18.3%, c50_(drug)_ = 18.6% contrast). Thresholds as assessed by AUROC were similar to the no flanker condition control condition, but they were significantly reduced for the drug condition (p = 0.004; threshold_(control)_ = 10.02%, threshold_(drug)_ = 5.76% contrast). Thus, in this instance mecamylamine caused flanker induced threshold reduction at flanker wavelength of 3*λ*.

**Figure 3:**
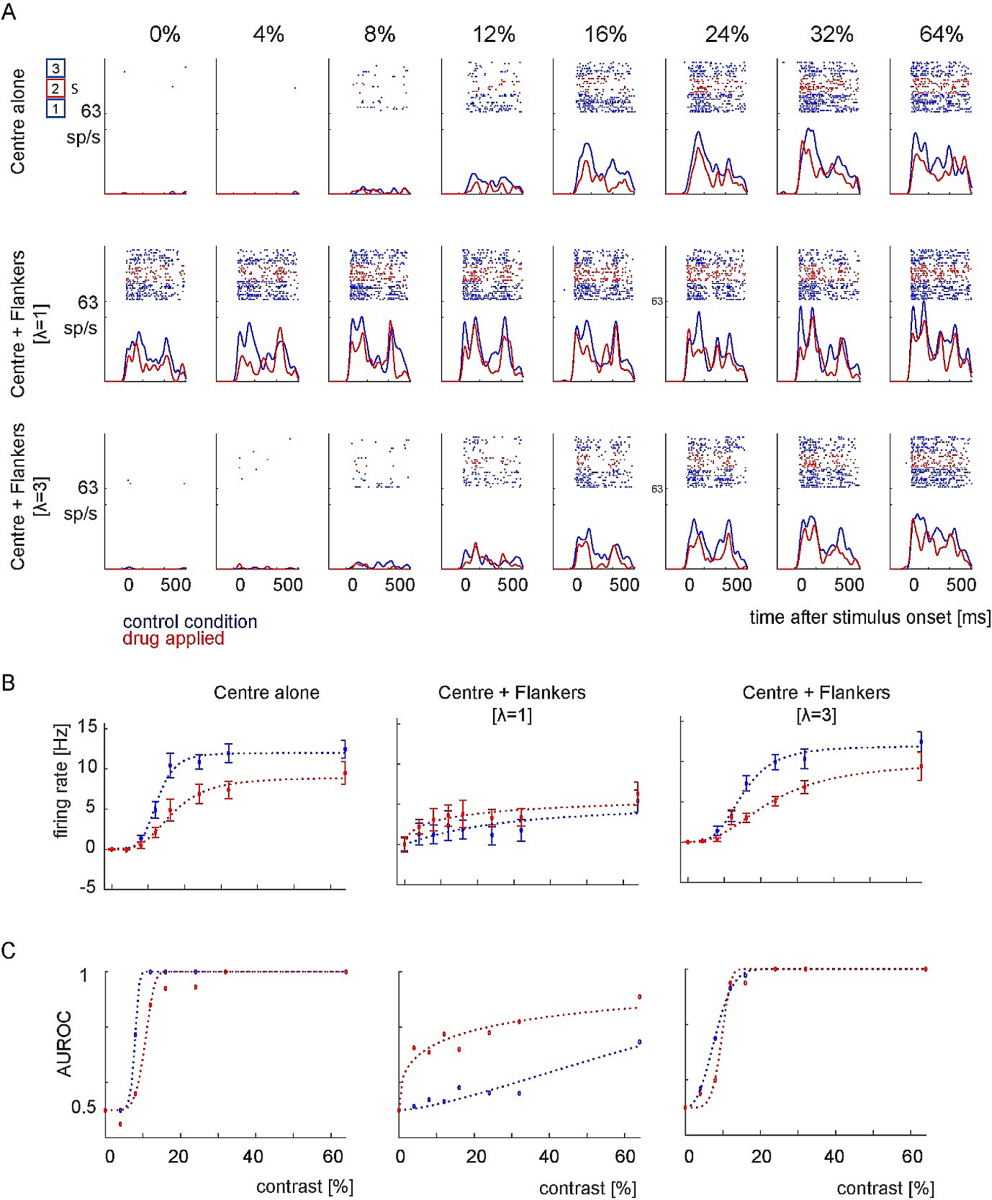
Effects of scopolamine on an example neuron. (**A**) All conventions as in Fig. 2A, except for S next to red box, which indicates that scopolamine was applied. The appearance of a central Gabor elicited a response that increased with the contrast of the stimulus. Scopolamine reduced firing rates. (**B**) Contrast *R*_*target*_ functions. When only the central Gabor was presented scopolamine caused a reduction in Rmax. This was also the case when the central Gabor was shown along with flankers of 3λ. However, for the flanker of 1λ condition Rmax was increased by scopolamine. (**C**) AUROC values showing the strength of flanker facilitation under control (blue) and scopolamine-applied (red) conditions. Scopolamine resulted in increased contrast thresholds when no flankers were shown and when flankers of 3λ were shown, but it decreased contrast thresholds when flankers of 1λ were shown.

Figure 3 illustrates the response of an example neuron under control conditions and when scopolamine was applied. The response depended significantly on stimulus contrast (F(7,1080) = 98.04, p < 0.001, 3 Factor ANOVA: contrast, drug, flankers), on drug application (F(1,1080) = 43.28, p < 0.001), and on the flanker presence (F(3,1080) = 94.06, p < 0.001). There was no drug*flanker interaction (F(3,1080) = 0.52, p = 0.671), or drug*contrast interaction (F(7,1080) = 1.65, p = 0.118), but there was a flankers * contrast interaction (F(21,1444) = 6.83, p < 0.001).

When two collinear flankers were presented without the central target (*R*_*Flankers*_), the neuron’s responses increased above the spontaneous activity level for flanker distances 1*λ*, but not 3*λ* (1*λ* and 3*λ*; *p* < 0.001 and *p* = 1.0 respectively, two-sided rank sum test). These effects did not depend on whether scopolamine was applied (drug *R*_*Flankers*_ vs spontaneous at 1*λ* and 3*λ*; *p* < 0.001 and *p* = 0.386, two-sided rank sum test). R_max_ was reduced for the center alone condition when the drug was applied (R_max(control)_ = 11.86 Hz, R_max(drug)_ = 8.95 Hz, Fig. 3B), and c50 increased with drug application (c50_(control)_ = 12.5%, c50_(drug)_ = 16.6%contrast). Thresholds as assessed by AUROC also changed significantly (p = 0.03; threshold_(control)_ = 2.38, threshold_(drug)_ = 5.63% contrast). At a flanker wavelength of 1*λ*, Rmax was overall reduced but this was less pronounced upon drug application (R_max(control)_ = 5.41 Hz, R_max(drug)_ = 6.96 Hz). C50 was increased for the control, but not the drug condition at a wavelength of 1*λ* (c50_(control)_ = 27.4%, c50_(drug)_ = 14.5%contrast). Thresholds were much higher compared to the no flanker condition, and differed significantly between control and drug condition (wavelength of 1*λ* p < 0.001; threshold_(control)_ = 88.7, threshold_(drug)_ = 29.33% contrast). At a flanker wavelength of 3*λ*, Rmax was similar to the no flanker condition, with little difference between control and drug application (R_max(control)_ = 11.91 Hz, R_max(drug)_ = 9.94 Hz). C50 were higher than the no flanker condition, especially for the drug condition (c50_(control)_ = 15.1%, c50_(drug)_ = 22.8%contrast). Thresholds were similar to the no flanker condition, and they were significantly increased for the drug condition (p = 0.037; threshold_(control)_ = 8.19%, threshold_(drug)_ = 11.31% contrast). Thus, in this instance scopolamine caused increased thresholds in the absence of flanker, reduced thresholds at flanker distances of 1*λ*, and increased thresholds at 3*λ*.

### Effects of collinear flankers and drugs on contrast response functions at the population level

**Figure 4:**
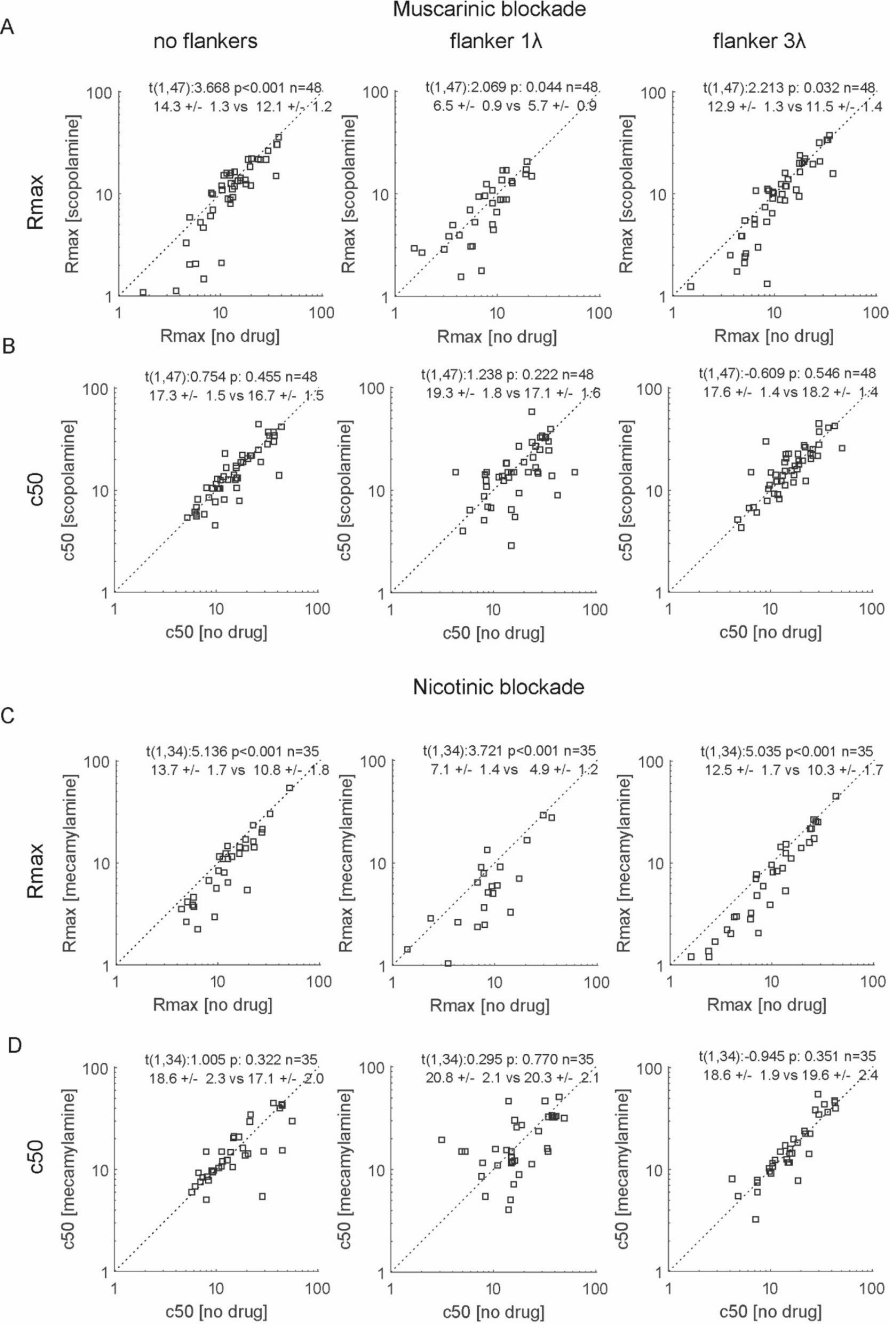
Effect of muscarinic and nicotinic blockade on Rmax and c50 at the population level for different flanker conditions. (**A**) Rmax in the no drug condition (x-axis) against scatterplot of Rmax of interest with scopolamine applied condition (y-axis). Left column shows results when no flankers were presented, middle column results for the flanker of 1λ, and right column shows results for the flanker of 3λ. Insets show paired t-test statistics along with the respective means ± S.E.M. of the distributions. (**B**) Same as A, but for slope instead of Rmax. (**C**) Same as **A**, but with mecamylamine as drug used. (**D**) same as B, but with mecamylamine as drug used.

These examples highlight that flankers can alter thresholds in a bi-directional way, and drug application can affect thresholds in a bi-directional way at the single cell level. Despite these variations, the effects at the population level were more homogenous. As previously shown^11,37^, we found that Rmax was significantly reduced by muscarinic and nicotinic blockade as well as by flanker presence (effect of scopolamine: F(1,47) = 11.0, p = 0.002, flanker effect F(2,94) = 54.4, p < 0.001, interaction F(2,94) = 3.1, p = 0.049; effect of mecamylamine: F(1,34) = 33.1, p < 0.001, flanker effect F(2,68) = 19.5, p < 0.001, interaction F(2,68) = 1.0, p = 0.382; repeated measures ANOVA). Figure 4 shows the effects of muscarinic and nicotinic blockade on the values of Rmax as derived from the Naka-Rushton fit for the no flanker condition, as well as the conditions when flankers of wavelength 1*λ* and 3*λ* were present. The change in Rmax and associated post-hoc test are given as inset. It shows that Rmax was reduced by drug application at all flanker wavelengths. Despite the fact that Rmax was significantly affected by drug application and by flanker presence/distance, c50 was not affected by either (Fig. 4; effect of scopolamine: F(1,47) = 1.1, p = 0.291, flanker effect F(2,94) = 0.5, p = 0.607, interaction F(2,94) = 1.2, p = 0.317; effect of mecamylamine: F(1,34) = 0.3, p = 0.615, flanker effect F(2,68) = 1.5, p = 0.287, interaction F(2,68) = 1.0, p = 0.382; repeated measures ANOVA).

### Effects of collinear flankers and drugs on neuronal sensitivity at the population level

**Figure 5:**
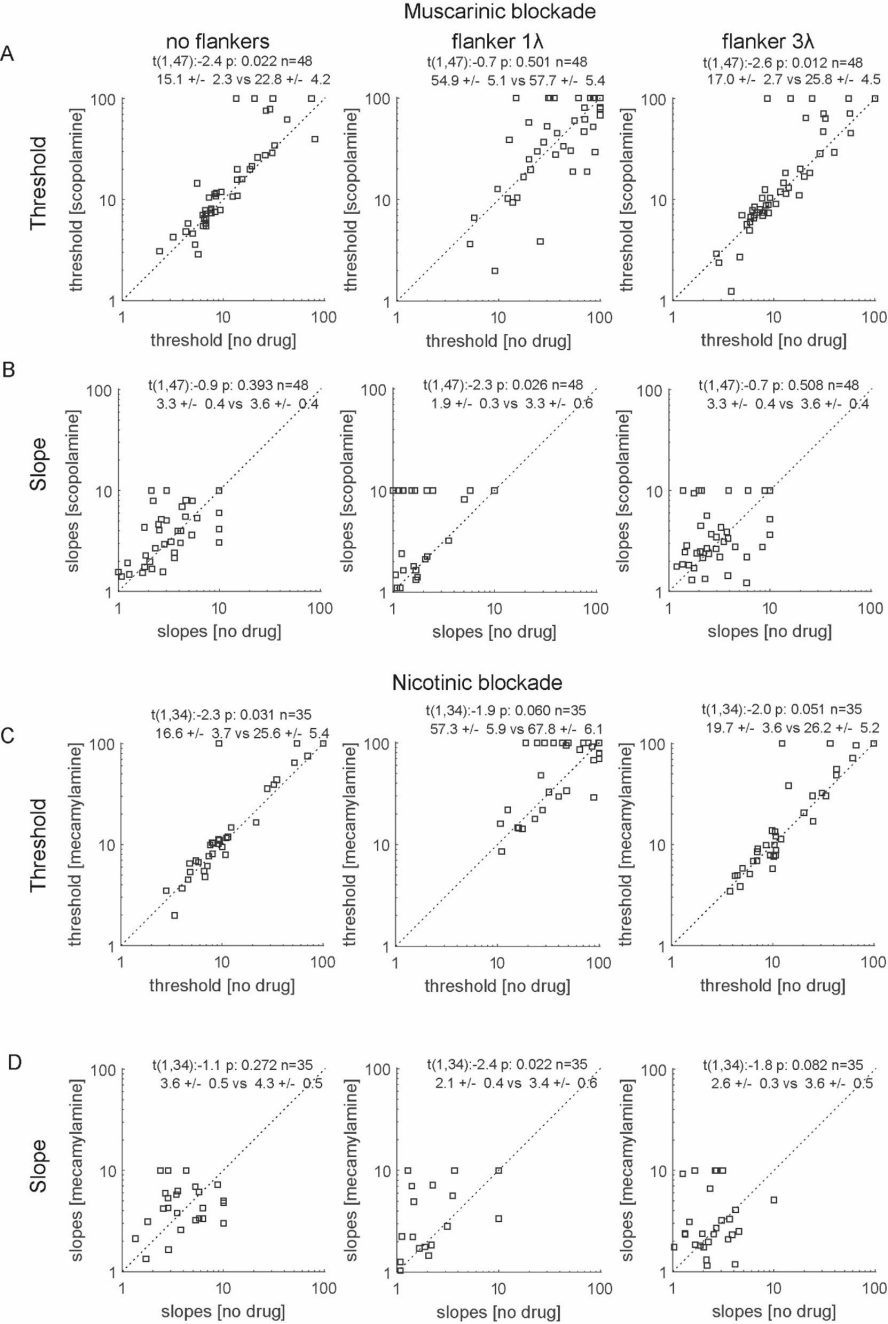
Effect of muscarinic and nicotinic blockade on neuronal thresholds and slopes at the population level for different flanker conditions. (**A**) Scatterplot of thresholds in the no drug condition (x-axis) against scatterplot of threshold in the drug applied (scopolamine) condition (y-axis). Left column shows results when no flankers were presented, middle column results for the flanker of 1λ, and right column shows results for the flanker of 3λ condition. Insets show paired t-test statistics along with the respective means ± S.E.M. of the distributions. (**B**) Same as **A**, but with slope instead of threshold as variable of interest. (**C,D**) same as **A** and **B**, but with mecamylamine instead of scopolamine used.

Rmax and c50 derived from the Naka-Rushton fit do not provide insight into changes of neuronal sensitivity, as changes in either measure may not translate into changes of neuronal thresholds. To assess the latter we calculated thresholds using Weibull fits to AUROC data. These fits reveal that drug application and flanker presence/distance changed neuronal thresholds (effect of scopolamine: F(1,47) = 5.7, p = 0.021, flanker effect F(2,94) = 59.7, p < 0.001, interaction F(2,94) = 1.2, p = 0.302; effect of mecamylamine: F(1,34) = 8.8, p = 0.005, flanker effect F(2,68) = 46.2, p < 0.001, interaction F(2,68) = 0.3, p = 0.761; repeated measures ANOVA). Slopes of contrast sensitivity were equally affected by drug application, while the effect of flankers on slopes was not significant (effect of scopolamine: F(1,47) = 4.3, p = 0.044, flanker effect F(2,94) = 1.8, p = 0.169, interaction F(2,94) = 1.8, p = 0.171; effect of mecamylamine: F(1,34) = 8.3, p = 0.006, flanker effect F(2,68) = 2.7, p = 0.169, interaction F(2,68) = 0.3, p = 0.759; repeated measures ANOVA). Overall, slopes were steeper with drug application (Fig. 5).

### Muscarinic and nicotinic effects on flanker induced facilitation and inhibition

**Figure 6:**
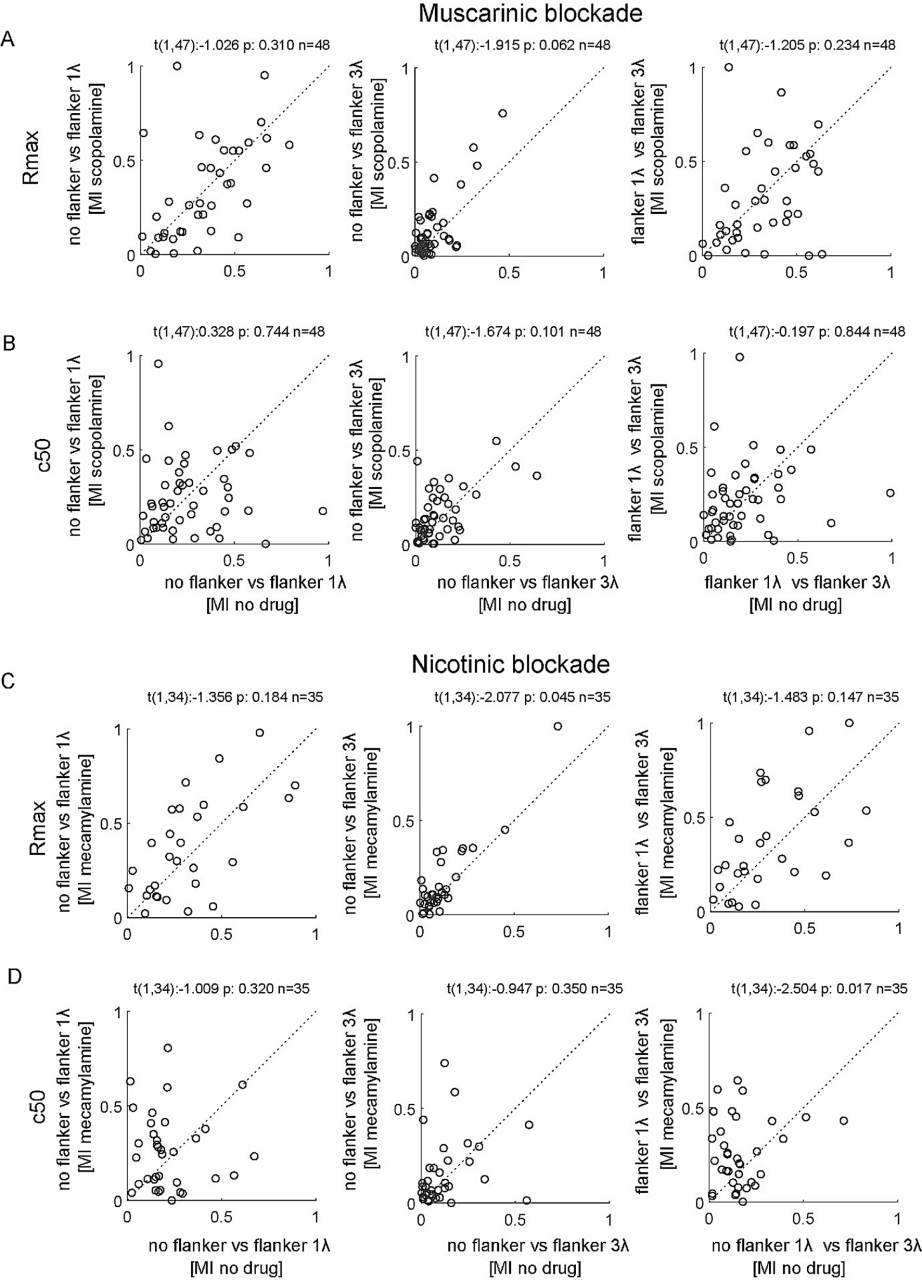
Effect of muscarinic and nicotinic blockade on flanker induced modulations of Rmax and c50. (**A**) Scatterplot of absolute values of modulation indices (MI) for Rmax in the no drug condition (x-axis) against scatterplot of absolute values of modulation indices (MI) for in the scopolamine applied condition (y-axis). Left column shows MIs for the no flanker versus flanker of 1λ condition, middle shows MIs for the no flanker versus flanker of 3λ condition, and right column shows MIs for the flanker of 3λ condition versus flanker of 1λ condition. Insets show paired t-test statistics. (**B**) Same as **A** but with absolute MIs for c50 values. (**C,D**) same as **A** and **B** but with mecamylamine as drug used, instead of scopolamine.

So far, we have shown that drug application and flanker presence affect neuronal sensitivity, but a key question of this study was whether the effects of flankers would be increased by scopolamine or by mecamylamine application. The former would be predicted based on the assumption that ACh reduced lateral cortical interactions by activating muscarinic receptors. Thus blocking these should increase lateral interactions, and thus should facilitate flanker effects. Equally, the effect of nicotinic blockade might be increased flanker effects, as it should reduce the feed-forward drive of the RF center stimulation, while having little or no effect on the lateral interactions, thus shifting the balance to increased flanker contribution. We addressed these question by calculating a flanker modulation index in the presence and in the absence of drug applied. Given that flanker effects could be positive as well as negative, the critical question was whether drug application would make it more (less) positive if it was positive in the first place, and whether it would make it more (less) negative if it was negative in the first place. If positive and negative flanker effects were evenly distributed, and the drug always increased the effect, the drug induced changes would cancel out at the population level, even though the basic hypothesis could prove correct. To address this possibility we took the absolute value of the Modulation index as the variable to investigate. We found that |Rmax MI| was not significantly affected by either drug (Fig. 6; effect of scopolamine: F(1,47) = 1.4, p = 0.301, flanker effect F(2,94) = 1.2, p = 0.300, interaction F(2,94) = 1.0, p = 0.361; effect of mecamylamine: F(1,34) = 3.9, p = 0.057, flanker effect F(2,68) = 11.6, p < 0.01, interaction F(2,68) = 0.8, p = 0.462; repeated measures ANOVA). There was no significant effect of either drug on |c50 MI| (effect of scopolamine: F(1,47) = 0.1, p = 0.749, flanker effect F(2,94) = 15.5, p < 0.001, interaction F(2,94) = 0.7, p = 0.514; effect of mecamylamine: F(1,34) = 3.4, p = 0.074, flanker effect F(2,68) = 9.2, p < 0.01, interaction F(2,68) = 1.2, p = 0.317; repeated measures ANOVA).

**Figure 7:**
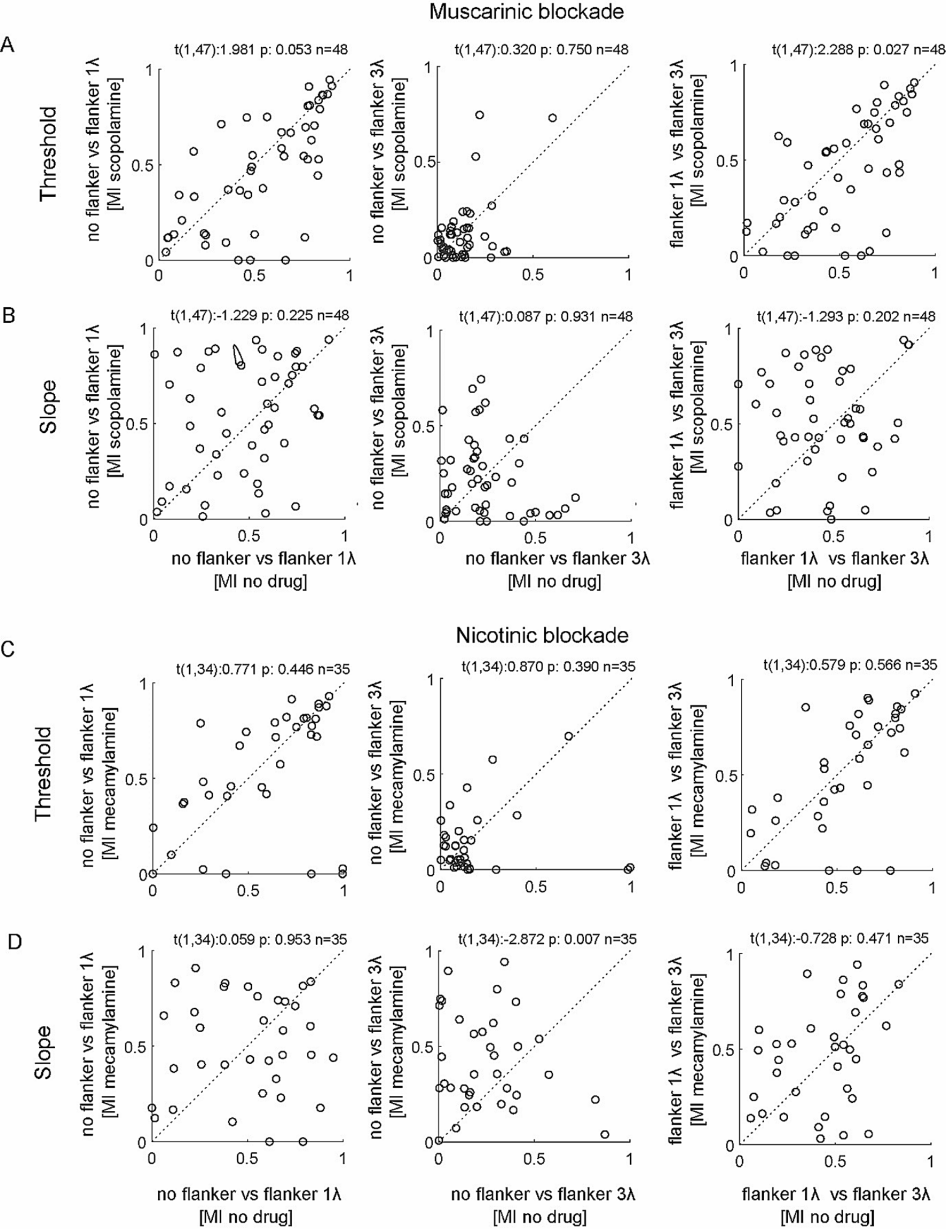
Effect of muscarinic and nicotinic blockade on flanker induced modulations of neuronal thresholds and slopes of contrast sensitivity function. (**A**) Scatterplot of threshold absolute values of modulation indices (MI) in the no drug condition (x-axis) against threshold absolute values of modulation indices (MI) for in the scopolamine applied condition (y-axis). Left column shows MIs for the no flanker versus flanker of 3λ condition, middle shows MIs for the no flanker versus flanker of 1λ condition, and right column shows MIs for the flanker of 3λ condition versus flanker of 1λ condition. Insets show paired t-test statistics. (**B**) Same as **A** but for absolute MIs for slope values. (**C,D**) Same as **A** and **B**, but with mecamylamine as drug employed.

However, there was a small but significant effect of muscarinic blockade on |threshold MI|, but not of nicotinic blockade (Fig. 7; effect of scopolamine: F(1,47) = 4.7, p = 0.034, flanker effect F(2,94) = 81.8, p < 0.001, interaction F(2,94) = 2.6, p = 0.078; effect of mecamylamine: F(1,34) = 0.7, p = 0.399, flanker effect F(2,68) = 52.6, p < 0.001, interaction F(2,68) = 0.1, p = 0.888; repeated measures ANOVA). When scopolamine was applied flanker effects were generally weaker (Fig. 7). No effect of drug application was found on the |slope MI| (effect of scopolamine: F(1,47) = 1.4, p = 0.250, flanker effect F(2,94) = 50.4, p < 0.001, interaction F(2,94) = 0.9, p = 0.406; effect of mecamylamine: F(1,34) = 2.8, p = 0.105, flanker effect F(2,68) = 9.1, p < 0.001, interaction F(2,68) = 3.5, p = 0.05; repeated measures ANOVA).

It might be possible that different effects occur during the transient response period, as this might be more dominated by feed-forward processing. To assess this possibility we determined |threshold MI| and |slope MI| for the period of 50–200 ms after stimulus onset. However, this did not yield a different outcome for either drug on |threshold MI| (effect of scopolamine: F(1,47) = 0.1, p = 0.806, flanker effect F(2,94) = 67.5, p < 0.001, interaction F(2,94) = 0.1, p = 0.931; effect of mecamylamine: F(1,34) = 0.0, p = 0.899, flanker effect F(2,68) = 33.8, p < 0.001, interaction F(2,68) = 0.6, p = 0.863; repeated measures ANOVA), or on |slope MI| (effect of scopolamine: F(1,47) = 0.2, p = 0.619, flanker effect F(2,94) = 33.2, p < 0.001, interaction F(2,94) = 0.3, p = 0.728; effect of mecamylamine: F(1,34) = 1.8, p = 0.183, flanker effect F(2,68) = 13.5, p < 0.001, interaction F(2,68) = 2.2, p = 0.117; repeated measures ANOVA).

Thus, our data show some, but limited support for the idea that ACh specifically affects contextual integration in macaque primary visual cortex through muscarinic receptor activation.

## Discussion

Collinear flankers generally caused reduced contrast sensitivity, irrespective of the center contrast. Muscarinic and nicotinic receptor blockade equally reduced contrast sensitivity at the population level. Contrary to our main hypothesis, neither muscarinic nor nicotinic receptor blockade systematically affected flanker induced contrast modulation, and the relatively small effects induced by muscarinic blockade resulted in reduced flanker interactions, rather than increased which is what we would have predicted. Our recordings were most likely confined to upper supragranular layers (Fig. 1C). We are thus unable to compare differences across layers for muscarinic versus nicotinic modulation, which would be predicted based on known differential receptor expression^34,35,51,52^. This biased sampling might also account for the finding that muscarinic receptor blockade shows some effect on contextual integration (the threshold MI), while nicotinic blockade did not show this effect.

Most of the cells recorded had parafoveal receptive fields, where magnocellular contributions dominate^53^. It might be the case that flanker facilitation occurs where parvocellular contributions dominate^7,54^, namely at foveal locations. Whether these would be differently affected by cholinergic modulation remains to be determined.

The effects of flanking stimuli on neuronal and perceptual contrast sensitivity have been studied extensively before. Some studies have suggested that collinear flankers cause facilitation of low contrast center stimuli, but inhibition of high contrast stimuli^13,21–23^, and that the low contrast induced facilitation translates into perceptual benefits^7,8,13,23^. Other studies have not been able to replicate these findings^11,55^, arguing instead that flankers on average result in reduced contrast sensitivity. Whether these discrepancies arise from differences in stimulus design or from differences in receptive field eccentricity^25^ is currently unknown. Our data presented here are in line with the results reporting flanker induced reduction in contrast sensitivity^11,55^.

Irrespective of the above mentioned discrepancies, we predicted that muscarinic receptor blockade should increase the influence of flanking stimuli on neuronal responses. This prediction is based on findings that demonstrate reduced intracortical interaction upon ACh release^30,32,56–59^, in conjunction with increased efficacy of feed-forward communication^30,34,35,56–58,60–63^. The reduced intra-cortical interactions are mediated by muscarinic (predominantly M2) receptors located on pre-synaptic terminals^30,33,56,57^. However, contrary to these predictions we did not find evidence for increased flanker interactions upon muscarinic blockade. In fact, the only significant effect of muscarinic blockade on flanker induced modulations was a reduced flanker modulation of contrast thresholds. None of the other flanker modulations were systematically affected. This contrasts with previous reports of altered spatial integration upon ACh application in primary visual cortex of non-human and human primates^32,63^. The discrepancy could arise from the type of drugs used. Both previous studies used a non-selective cholinergic agonist, thereby stimulating muscarinic and nicotinic receptors, while we used a muscarinic antagonist. It might be the combined effect on muscarinic and nicotinic receptors that drove the altered spatial integration in the previous studies. Alternatively, these differences could also result from differences in stimuli used. Roberts et al. (2005) measured spatial summation using continuous orientated bars, while our stimuli were non-continuous. Whether or not a stimulus is continuous or not, along with the spatial distancing, may affect to what extent surround suppression effects are mediated predominantly by lateral interactions in V1, or by feedback interactions from higher cortical areas, or a mixture of the two^14–16,64–68^. If that is the case, our results could suggest that feedback terminals exhibit a different expression pattern of muscarinic receptors than lateral intra-areal connections in non-human primates. Alternatively feedback from different areas could signal different aspects of a scene, and also show differential muscarinic expressions pattern as present in mouse V1^69,70^. Either possibility require future investigation.
